# Potentials of genotypes, morpho-physio-biochemical traits, and growing media on shelf life and future prospects of gene editing in tomatoes

**DOI:** 10.3389/fgeed.2023.1203485

**Published:** 2023-08-23

**Authors:** Renu Yadav, Sarika Jaiswal, Tripti Singhal, Rohit Kumar Mahto, S. B. Verma, Ramesh Kumar Yadav, Rajendra Kumar

**Affiliations:** ^1^ Amity Institute of Organic Agriculture (AIOA), Noida, Uttar Pradesh, India; ^2^ Division of Bioinformatics, Indian Agricultural Statistics Research, Institute, New Delhi, India; ^3^ Division of Genetics, IARI, New Delhi, India; ^4^ Division of Vegetable Science, IARI, New Delhi, India

**Keywords:** gene editing, organic farming, shelf life, traditional farming, tomato

## Abstract

**Background:** To study the genetic basis of the impact of genotypes and morpho-physio-biochemical traits under different organic and inorganic fertilizer doses on the shelf life attribute of tomatoes, field experiments were conducted in randomized block designs during the rabi seasons of 2018–2019 and 2019–2020. The experiment comprised three diverse nutrient environments [T1—organic; T2—inorganic; T3—control (without any fertilizers)] and five tomato genotypes with variable growth habits, specifically Angoorlata (Indeterminate), Avinash-3 (semi-determinate), Swaraksha (semi-determinate), Pusa Sheetal (semi-determinate), and Pusa Rohini (determinate).

**Results:** The different tomato genotypes behaved apparently differently from each other in terms of shelf life. All the genotypes had maximum shelf life when grown in organic environments. However, the Pusa Sheetal had a maximum shelf life of 8.35 days when grown in an organic environment and showed an increase of 12% over the control. The genotype Pusa Sheetal, organic environment and biochemical trait Anthocyanin provides a promise as potential contributor to improve the keeping quality of tomatoes.

**Conclusion:** The genotype Pusa Sheetal a novel source for shelf life, organic environment, and anthocyanin have shown promises for extended shelf life in tomatoes. Thus, the identified trait and genotype can be utilized in tomato improvement programs. Furthermore, this identified trait can also be targeted for its quantitative enhancement in order to increase tomato shelf life through a genome editing approach. A generalized genome editing mechanism is consequently suggested.

## Introduction

The tomato (*Solanum Lycopersicon* L) is an herbaceous plant grown annually for its nutritional content. Globally, tomato is a chief cultivated as well as an expended vegetable crop with *per capita* ingesting of either fresh or by products quantity of about 21 kg or aproximately19% of the total vegetable consumption per year (FAOSTAT, 2020). It is one of the most inexpensive and readily accessible reservoirs of proteins, minerals, vitamins, and essential amino acids (Stephen et al., 2014), and is considered to be very rich in antioxidants and biologically active compounds such as phenolics, flavonoids, beta-carotene, and lycopene that help as endogenous defense mechanisms produced in response to pathogens (Simova-Stoilova et al., 2006; Bhowong et al., 2009; Pinela et al., 2012). Lycopene, which is contained in ripe tomatoes, is an antioxidant that can lead to defense against carcinogenic components. Carotenoid lycopene, one of the most important antioxidants, has been associated with reduced risk of numerous forms of cancer and heart disease (Adeniyi and Ademoyegun, 2012). Organically grown tomatoes have been found to have a significant impact on nutrient composition as compared to tomato cultivation through conventional fertilizers (Shankar et al., 2012). Organic farming has demonstrated improved nutritional properties in fruits and vegetables through several studies (Luthria et al., 2010). A relative learning showed that organic tomato juice had more phenolics and hydrophilic antioxidant activity when compared to conventionally grown tomato soup (Vallverdu et al., 2012).

The use of organic fertilizers plays a major role in confirming the sustainability of production, allowing the protection of original supplies for current and future generations, while providing high quality and an extended shelf life (Rembiałkowska, 2007). The addition of organic manure to the soil augments microbial activity, rises its ability to conserve fertilizer, and improves fertility and fertilizer use efficiency as an end goal (Nanwai et al., 1998). Substantial amounts of available organic material, for example, farmyard manure, poultry waste, and peat fertilizer, should be considered as alternative and economical sources of fertilizer. In addition, organic fertilizers can act as an energy source for microbes present in the soil, which can improve soil composition and plant growth. Efforts to reduce the undesirable impacts of native rock fertilizers on the environment and the escalating demand of customers for tomato fruit due to its nutritional benefits have stimulated scientists and growers to develop the means to meet the requirements for extended shelf life. The current study aims to evaluate the effects of morpho-physio-biochemical traits, organic-inorganic nutrient sources, and identify the best-performing tomato varieties in terms of shelf life.

## Materials and methods

### Experimental site

Farm experimentation was carried out at Amity University, Noida, Uttar Pradesh, during the period 2018–2019 and 2019–2020. Geographically, the experimental farm is situated at 28.530 north latitude and 77.390 east longitude, at a height of 202 m above sea level. This agroecological region has a hot and sub-humid (dry) climate. The soil of the experimental site consisted of sandy loam with low organic carbon content and was alkaline in nature. The physical and chemical characteristics of the soil in the experimental field were: pH 7.8, EC 0.528 mS cm^−1^, organic carbon 0.58%, available nitrogen—263.24 kg ha^−1^, available phosphorus—25.9 kg ha^−1^, and available potassium—332.8 kg ha^−1^.

### Agronomic practices, management, and treatment details

The experiment, consisting of three diverse nutritional environments [T1–organic; T2—inorganic; T3—control (no fertilizer)] and five tomato varieties (Table 1) with varied growth habits collected from different institutions, namely, Angoorlata (V1), Avinash-3 (V2), Pusa Rohini (V3), Pusa Sheetal (V4), and Swaraksha (V5), was conducted in a randomized block design with three replications. Each plot was 4 × 4 m in size. The seedbed was grown on a raised bed, and plantlets were relocated after 4 weeks when the seedlings were 9–12 cm in length with four to six compound leaflets. The organic fertilizer consisted of farmyard manure, vermicompost at 15-20 *t*/ha at the time of the last plowing, and dhaincha (*Sesbania aculeate* L) green manure, which was incorporated in the soil crust. Inorganic fertilizers: 100–125 kg nitrogen fertilizer (urea), 60–80 kg P_2_O_5_ (single super phosphate), and 40–50 kg K_2_O (muriate of potash). In total, 1/3 N, the whole of P_2_O_5_ and K_2_O were mixed in the soil at the time of transplanting, and the remaining nitrogen fertilizer was applied 45 days after transplanting.

**TABLE 1:** Details of tomato varieties.

S. No.	Tomato variety	Source of collection	Growth habit
1	Pusa Sheetal	ICAR- Indian Agricultural Research Institute, New Delhi, India	Semi determinate
2	Pusa Rohini	ICAR- Indian Agricultural Research Institute, New Delhi, India	Determinate
3	Angoorlata	ICAR-IIVR—Indian Institute of Vegetable Research, Varanasi, India	Indeterminate
4	Swaraksha	ICAR-IIVR—Indian Institute of Vegetable Research, Varanasi, India	Semi Determinate
5	Avinash 3	Syngenta India Pvt. Ltd	Semi Determinate

### Soil analysis

Soil samples were collected from the field by scraping away the topsoil and making a V-shaped cut using a spade or a khurpa to a depth of 6 inches and 1 thick soil layer from one side of the V shape cut was collected as soil sample. Similarly, soil samples were collected from seven/eight locations in the field of uniform texture and uniform fertility and were dried in the shade. The physicochemical characteristics of the soil for pH, EC, organic carbon (Olsen et al., 1954), nitrogen (Subbiah and Asija, 1956), phosphorus (Walkley and Black, 1934), and potassium (Perur et al., 1973) were analyzed according to the indicated procedures.

### Observations recorded on shelf life

PH = Plant height (cm), NB = Number of Branches, NL = Number of Leaves, NF = Number of Flowers, NFr = Number of Fruits, FrL = Fruit Length (cm), FrD = Fruit Diameter (cm), FrW = Fruit Weight (g), SL Ref T = Shelf Life at Refrigerated Temperature (days), Ly = Lycopene (mg/100 gm fresh weight), TPC = Total phenolic content (mg CA/g dry weight), An = Anthocyanin (mg/100 g fresh weight), Tot Vit C = Total Vitamin C (mg/100 g fresh weight), AP = Ascorbate peroxidase (moles H2O2 reduced min^−1^ g^−1^ fresh wt), SOD = Superoxide dismutase (U/mg protein), PAL = Phenylalanine ammonia-lyase PAL (µmol/t-ca/mg protein/h), SL RT = Shelf Life at Refrigerated Temperature (days).

The shelf life (days) of fruit at room and refrigerated temperatures was documented for five fruits/plants/genotypes. The shelf life was determined on the basis of the time elapsed between the time of picking and the start of fruit decay. The parameters studied were fruit wrinkling and watery days. The harvested, ripe fruits were kept on a clean table in a laboratory at room temperature (24–30°C) to observe the critical variations on a daily basis. Symptoms on watery, wrinkleless and wrinkled fruits were observed for several days and documented as unit in number of days.

### Analysis of phenotypic data

The data were recorded for tomato shelf-life at room and refrigerator temperatures, as shown in Table 2. The mean values of the five observations were calculated for all characteristics. The gathered data were subjected to biometric analysis using R software (version 4.2.0**).** The values of genotypic vs phenotypic coefficients of variation were estimated as per Burton (1953) and classified (<10% = low, 10%–20% = moderate, and >20% = high) as suggested by Sivasubramanian and Menon (1973). Heritability in the broader sense was obtained using the formula proposed by Allard (1960) and categorized (with 0%–30% = low, 31%–61% = medium, 61%–100% = high) as suggested by Robinson et al. (1949). The expected genetic advance as a percentage of the mean was calculated and categorized as low (<10%), moderate (10%–20%), and high (>20%), as suggested by Johnson et al. (1955). The covariance values for all possible combinations of traits were calculated as per Panse and Sukhatme (1978). The correlation coefficients were calculated according to Miller et al. (1958). The path analysis was performed as suggested by Wright (1921) and elaborated according to Wright (1960) and Dewey and Lu (1959). Heat maps and principal component analysis were performed using R with the *factoextra* package (https://cran.r-project.org/web/packages/factoextra/index.html).

**TABLE 2:** Pooled increase/Decrease (%) of tomato shelf life over control and general statistics.

Variety and general tatistics	Shelf life					
	Room temperature			Refrigerated temperature		
	T1	T2	T3	T1	T2	T3
V1	7.7 ± 0.03 (−14%)	6.46 ± 0.13 (3%)	6.65 ± 0.35	13.16 ± 0.18 (−7%)	12.65 ± 0.02 (−4%)	12.18 ± 0.51
V2	7 ± 1.33 (−16%)	6.35 ± 1.02 (−8%)	5.85 ± 0.85	9.35 ± 2.35 (−20%)	8.51 ± 2.17 (−12%)	7.5 ± 1.16
V3	7.84 ± 0.16 (−13%)	6.83 ± 0.17 (0%)	6.81 ± 0.19	11.17 ± 0.17 (−9%)	10.65 ± 0.02 (−5%)	10.17 ± 0.17
V4	8.5 ± 0.5 (−12%)	8.35 ± 0.02 (−10%)	7.5 ± 0.5	14.81 ± 0.52 (−20%)	12.65 ± 0.02 (−6%)	11.84 ± 0.5
V5	7.5 ± 1.5 (−11%)	6.84 ± 0.84 (−2%)	6.69 ± 0.68	9.34 ± 0.34 (−11%)	8.84 ± 0.16 (−6%)	8.31 ± 0.03
Mean	7.124			10.740		
Range	5–9.990			6–16		
CD at 5% for Varieties	1.240			2.411		
CD at 5% for Treatments	0.583			1.207		
SE	0.226			0.254		
*R* ^2^	0.505			0.742		
CV (%)	12.91			12.037		
GCV (%)	12.54			20.595		
PCV (%)	15.67			21.93		
Heritability (BS) (%)	64.00			86.00		
Genetic Advance (mean%)	21.18			39.95		

V1, Angoorlata; V2, Avinash3; V3, Pusa Rohini; V4, Pusa Sheetal; V5, swaraksha.

## Results

### Analysis of variance

The ANOVA revealed that the shelf life of tomatoes at room and refrigerated temperature conditions was significantly affected by varietal differences, the application of organic and inorganic nutrient sources, and environmental variations (Table 3).

**TABLE 3:** Analysis of variance (ANOVA) for tomato shelf life.

Variables	Sources of variation	Mean Sum of Square	F value	Pr > F
Shelf life at Room Temperature	Year	13.705	16.19	0.0001
	Treatment	8.164	9.65	0.0002
	Variety	7.039	8.32	<.0001
Shelf life at Refrigerated Temperature	Year	9.069	5.43	0.0223
	Treatment	18.575	11.12	<.0001
	Variety	81.769	48.93	<.0001

### Effect of different factors on tomato shelf life

**FIGURE 1:**
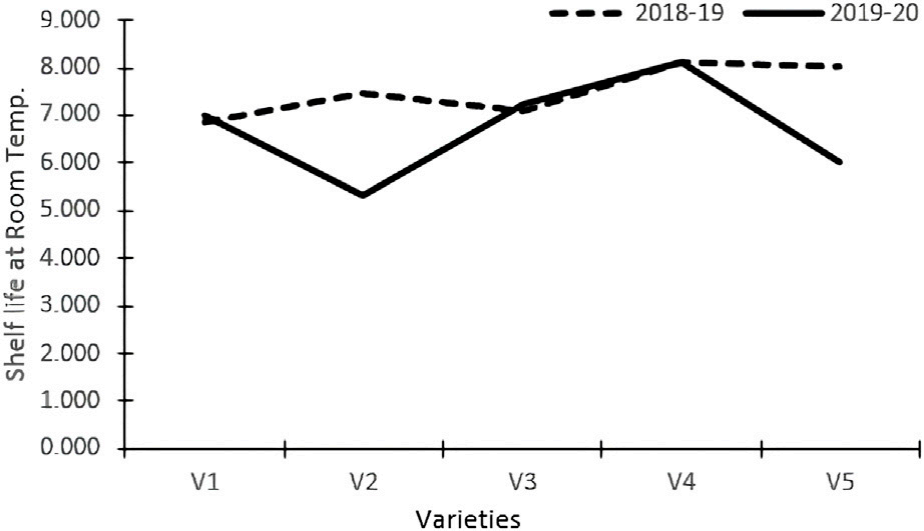
Shelf-life room temperature.

Varieties and treatments applied had a significant effect on the shelf-life of tomatoes at room temperature (Table 3). The tomato fruits were stored at room temperature and visually inspected. The average number of days that tomato fruits took to wrinkle was higher in organically grown environments than in inorganic environments. All the genotypes had maximum shelf life when grown in an organic environment. However, Pusa Sheetal had a maximum shelf-life of 8.35 days when grown in an organic environment, showing an increase of 12% over the control (Table 2; Figure 1). The Swaraksha variety did not reflect any difference in behavior for shelf life with respect to organic and inorganic environments. Overall, an organic environment resulted in a longer shelf-life at room temperature.

**FIGURE 2:**
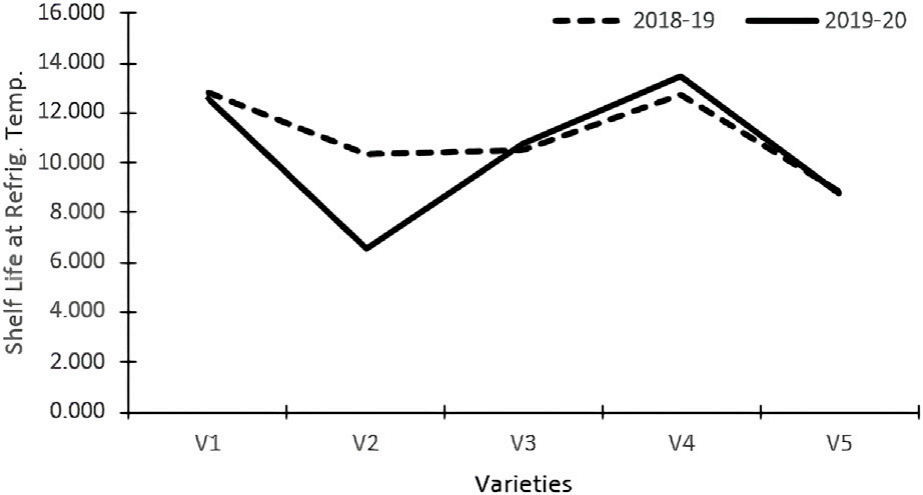
Shelf-life refrigerated temperature.

The varieties and their interactions with treatments had a significant influence on the keeping quality of tomato fruits at refrigerated temperatures (Table 3). The fruits were stored in refrigerated conditions (4–8°C) and evaluated visually. The keeping quality of all the genotypes was better in the organic environment as compared to the inorganic environment. The best shelf-life was documented at 14.80 days for the tomato variety Pusa Sheetal, followed by Angoorlata (13.16 days) and Pusa Rohini (11.17 days), respectively, and presented in Table 2 and Figure 2.

### Coefficient of variation, heritability, and genetic advance

The shelf life of tomatoes at room temperature, with a range of 5–9.99 and a mean of 7.12, expressed considerable variations in terms of genotypic coefficient (12.54%), phenotypic coefficient (15.57%), coefficient of variation (12.91%), heritability in the broad sense (64%), and genetic advance (21.18%). These data are presented in Table 2. However, the shelf life of tomato fruit at refrigerated temperature, with a range of 6–16 and mean of 10.74 expressed considerable variations as a genotypic coefficient (20.59%), phenotypic coefficient (21.93%), coefficient of variation (12.04%), heritability in the broad sense (86%), and genetic advance (39.95%). These data are also presented in Table 2.

## Correlation coefficients, associations between different component factors, and path analysis

The pooled analysis of the shelf life of tomato fruits stored at room temperature revealed positive and significant phenotypic correlation coefficient values with fruit length (0.36), fruit weight (0.50), lycopene (0.36), refrigeration temperature (0.55), anthocyanins (0.33), and phenylalanine ammonia-lyase (0.41). However, the pooled ANOVA for the shelf life of tomato fruits stored under refrigerated conditions had highly significant and positive correlation coefficient values with traits such as plant height (0.28), number of leaves (0.46), total phenolic content (0.48), and anthocyanins (0.32), but negative and significant values with traits such as number of fruits (−0.32) and total vitamin C (−0.40). However, in general, for almost all traits, genotypic correlation coefficient values for tomato shelf life at room temperature and refrigerated room temperature were found to be higher than their respective phenotypic values.

The associations between different combinations of factors for all the parameters studied in the current investigation during the years 2018–19 and 2019–20 and combined for both periods were presented as color gradients through heat maps, as shown in Supplementary Figures S1–S3, respectively.

The path analysis as presented in Table 4 revealed that the genotypic correlation for shelf life of tomato fruits stored at room temperature environment was positively influenced by the trait shelf life at refrigerated temperature (1.42), followed by FrW (1.15), total vitamin C (0.91), phenylalanine ammonia-lyase (0.71), BN (0.51), FrL (0.38), PHt (0.30), and ascorbate peroxidase (0.08). However, the trait FrD (−1.15) contributed the maximum direct negative effect, followed by Ly (−0.88), An (−0.72), NL (−0.27), TPC (−0.23), NF (−0.18), SOD (−0.15), and FrN (−0.03). On the other hand, the path analysis as presented in Table 5 indicated that the genotypic correlation of shelf life of tomato fruits stored at refrigerated temperature was positively influenced by the trait An (0.75), followed by FrD (0.71), Ly (0.58), SL RT (0.56), and NL (0.51). However, the trait FrW (−1.12) contributed the maximum direct negative effect, followed by PAL (−0.46), SOD (−0.35), PHt (−0.33), and NF (−0.25).

**TABLE 4:** Pooled Path Analysis (Direct and indirect effects) Matrix for Tomato Shelf Life at Room Temperature.

Tr→↓	PH	BN	LN	FN	Fr N	FrL	FrD	FrW	SL Ref T	Ly	TPC	An	Tot Vit C	AP	SOD	PAL	SL R T
**PH**	**0.30**	0.18	0.19	0.20	0.04	0.10	0.07	−0.04	0.12	0.06	0.08	−0.08	−0.06	0.06	0.03	−0.09	0.16
**BN**	0.31	**0.51**	0.47	0.40	−0.05	−0.05	−0.13	−0.12	0.19	−0.01	0.41	0.04	−0.28	0.45	0.24	−0.06	−0.01
**LN**	−0.17	−0.25	**−0.27**	−0.20	0.05	0.04	0.02	0.02	−0.18	−0.08	−0.23	−0.01	0.15	−0.21	−0.04	0.01	0.16
**FN**	−0.12	−0.14	−0.13	**−0.18**	−0.07	−0.05	−0.05	−0.02	−0.02	−0.07	−0.07	0.01	0.003	−0.12	−0.08	−0.04	0.01
**Fr N**	−0.00	0.00	0.01	−0.01	**−0.03**	−0.03	−0.03	−0.02	0.01	−0.02	0.02	−0.01	−0.03	−0.01	−0.02	−0.01	0.15
**FrL**	0.13	−0.04	−0.06	0.11	0.33	**0.38**	0.35	0.24	0.01	0.24	−0.18	0.04	0.28	0.01	0.15	0.18	0.51
**FrD**	−0.26	0.29	0.08	−0.30	−0.95	−1.07	**−1.15**	−0.82	0.06	−0.90	0.59	0.12	−0.96	−0.20	−0.39	−0.60	0.44
**FrW**	−0.17	−0.28	−0.11	0.10	0.80	0.74	0.82	**1.15**	0.18	0.87	−0.31	0.74	0.73	0.02	0.26	0.79	0.58
**SL RefT**	0.56	0.52	0.92	0.18	−0.52	0.02	−0.07	0.22	**1.42**	0.38	0.84	0.47	−0.72	0.38	−0.24	0.00	0.77
**Ly**	−0.19	0.02	−0.25	−0.37	−0.46	−0.56	−0.69	−0.66	−0.23	**−0.88**	0.10	−0.14	−0.51	−0.39	−0.22	−0.74	0.64
**TPC**	−0.06	−0.18	−0.19	−0.09	0.12	0.11	0.12	0.06	−0.13	0.03	**−0.23**	−0.05	0.18	−0.10	−0.00	0.03	0.06
**An**	0.21	−0.06	−0.02	0.05	−0.13	−0.07	0.07	−0.47	−0.24	−0.11	−0.17	**−0.72**	0.03	−0.03	−0.09	−0.22	0.56
**Tot Vit C**	−0.17	−0.50	−0.50	−0.02	0.76	0.67	0.76	0.58	−0.46	0.54	−0.72	−0.03	**0.91**	0.00	0.34	0.57	0.11
**AP**	0.02	0.07	0.06	0.05	0.03	0.00	0.01	0.00	0.02	0.03	0.03	0.00	0.00	**0.08**	0.08	0.04	0.19
**SOD**	−0.01	−0.07	−0.02	−0.07	−0.10	−0.06	−0.05	−0.03	0.02	−0.04	−0.00	−0.02	−0.05	−0.14	**−0.15**	−0.06	0.19
**PAL**	−0.21	−0.09	−0.02	0.13	0.32	0.33	0.37	0.48	0.00	0.60	−0.10	0.21	0.44	0.37	0.31	**0.71**	0.51
**rg with SL R T**	0.16	−0.01	0.16	0.01	0.15	0.51	0.44	0.58	0.77	0.64	0.06	0.57	0.11	0.19	0.19	0.51	**1.0000**
**Par. r2**	0.05	−0.00	−0.04	−0.00	−0.00	0.19	−0.50	0.67	1.09	−0.56	−0.01	−0.41	0.10	0.01	−0.03	0.36	-

Underlined Values indicate direct effects.

Rg = genotypic correlation coefficient, PH, Plant height (cm); NB, number of branches; NL, number of leaves; NF, number of flowers; NFr, Number of Fruits; FL, Fruit Length (cm); FD, Fruit Diameter (cm); FW, Fruit Weight (g); SL, Ref T = Shelf Life at Refrigerated Temperature (days); Ly, Lycopene (mg/100 gm fresh weight); TPC, Total phenolic content (mg CA/g dry weight); An, Anthocyanin (mg/100 g fresh weight); Tot Vit C = Total Vitamin C (mg/100 g fresh weight), AP, Ascorbate peroxidase (moles H2O2 reduced min-1 g-1 fresh wt), SOD, Superoxide dismutase (U/mg protein); PAL, Phenylalanine ammonia-lyase PAL (µmol/t-ca/mg protein/h), SL RT, Shelf Life at Refrigerated Temperature (days).

**TABLE 5:** Pooled Path Analysis (Direct and indirect effects) Matrix for Tomato Shelf Life at Refrigerated Temperature.

Tr→↓	PH	BN	LN	FN	Fr N	FrL	FrD	FrW	SL RT	Ly	TPC	An	Tot Vit C	AP	SOD	PAL	SL Ref T
**PH**	**−0.33**	−0.20	−0.21	−0.22	−0.05	−0.11	−0.07	0.05	−0.05	−0.07	−0.09	0.09	0.06	−0.06	−0.03	0.09	0.40
**BN**	0.08	**0.13**	0.12	0.11	−0.01	−0.01	−0.03	−0.03	−0.00	−0.00	0.11	0.01	−0.07	0.12	0.06	−0.02	0.37
**LN**	0.32	0.47	**0.51**	0.38	−0.10	−0.08	−0.04	−0.05	0.08	0.14	0.43	0.01	−0.28	0.38	0.07	−0.02	0.67
**FN**	−0.16	−0.19	−0.18	**−0.24**	−0.09	−0.07	−0.06	−0.02	−0.00	−0.10	−0.09	0.02	0.00	−0.16	−0.11	−0.04	0.13
**Fr N**	0.00	−0.00	−0.00	0.00	**0.01**	0.01	0.01	0.01	0.00	0.01	−0.01	0.00	0.01	0.00	0.01	0.00	−0.36
**FrL**	0.02	−0.06	−0.01	0.02	0.05	**0.06**	0.05	0.04	0.03	0.04	−0.03	0.00	0.04	0.00	0.02	0.03	0.02
**FrD**	0.16	−0.18	−0.05	0.18	0.59	0.66	**0.71**	0.50	0.31	0.55	−0.36	−0.07	0.59	0.12	0.24	0.37	−0.05
**FrW**	0.17	0.27	0.10	−0.10	−0.78	−0.72	−0.80	**−1.12**	−0.65	−0.84	0.30	−0.72	−0.71	−0.02	−0.25	−0.76	0.16
**SL RT**	0.09	−0.00	0.09	0.01	0.08	0.28	0.24	0.32	**0.56**	0.35	0.03	0.31	0.06	0.10	0.10	0.28	0.77
**Ly**	0.12	−0.01	0.17	0.25	0.30	0.37	0.46	0.44	0.37	**0.58**	−0.07	0.09	0.34	0.26	0.15	0.49	0.26
**TPC**	−0.01	−0.02	−0.02	−0.01	0.01	0.01	0.01	0.01	−0.00	0.00	**−0.02**	−0.01	0.02	−0.01	−0.00	0.00	0.59*
**An**	−0.21	0.06	0.02	−0.06	0.14	0.07	−0.07	0.49	0.42	0.12	0.18	**0.75**	−0.03	0.03	0.10	0.22	0.33
**Tot Vit C**	0.02	0.07	0.07	0.00	−0.11	−0.10	−0.11	−0.09	−0.01	−0.08	0.11	0.00	**−0.13**	−0.00	−0.05	−0.08	−0.51
**AP**	0.02	0.07	0.06	0.05	0.03	0.00	0.01	0.00	0.01	0.03	0.03	0.00	0.00	**0.08**	0.08	0.04	0.27
**SOD**	−0.03	−0.16	−0.05	−0.16	−0.23	−0.14	−0.12	−0.08	−0.06	−0.09	−0.00	−0.04	−0.13	−0.33	**−0.35**	−0.15	−0.16
**PAL**	0.13	0.06	0.01	−0.08	−0.21	−0.21	−0.24	−0.31	−0.23	−0.39	0.07	−0.14	−0.29	−0.24	−0.20	**−0.46**	0.00
**rg with SL R T**	0.40	0.37	0.65	0.13	−0.36	0.02	−0.05	0.16	0.77	0.26	0.59	0.33	−0.51	0.27	−0.16	0.00	**1.00**
**Par. r2**	−0.13	0.05	0.33	−0.03	−0.00	0.00	−0.04	−0.17	0.44	0.15	−0.01	0.25	0.07	0.02	0.06	−0.00	-

Underlined Values indicate direct effects.

Rg = genotypic correlation coefficient; PH, Plant height (cm); NB, number of branches; NL, number of leaves; NF, number of flowers; NFr, Number of Fruits; FL, Fruit Length (cm); FD, Fruit Diameter (cm); FW, Fruit Weight (g); SL, Ref T = Shelf Life at Refrigerated Temperature (days); Ly = Lycopene (mg/100 gm fresh weight); TPC, Total phenolic content (mg CA/g dry weight); An = Anthocyanin (mg/100 g fresh weight); Tot Vit C = Total Vitamin C (mg/100 g fresh weight); AP, Ascorbate peroxidase (moles H2O2 reduced min-1 g-1 fresh wt); SOD, Superoxide dismutase (U/mg protein); PAL, Phenylalanine ammonia-lyase PAL (µmol/t-ca/mg protein/h); SL RT, Shelf Life at Refrigerated Temperature (days).

### Principal component analysis

**FIGURE 3:**
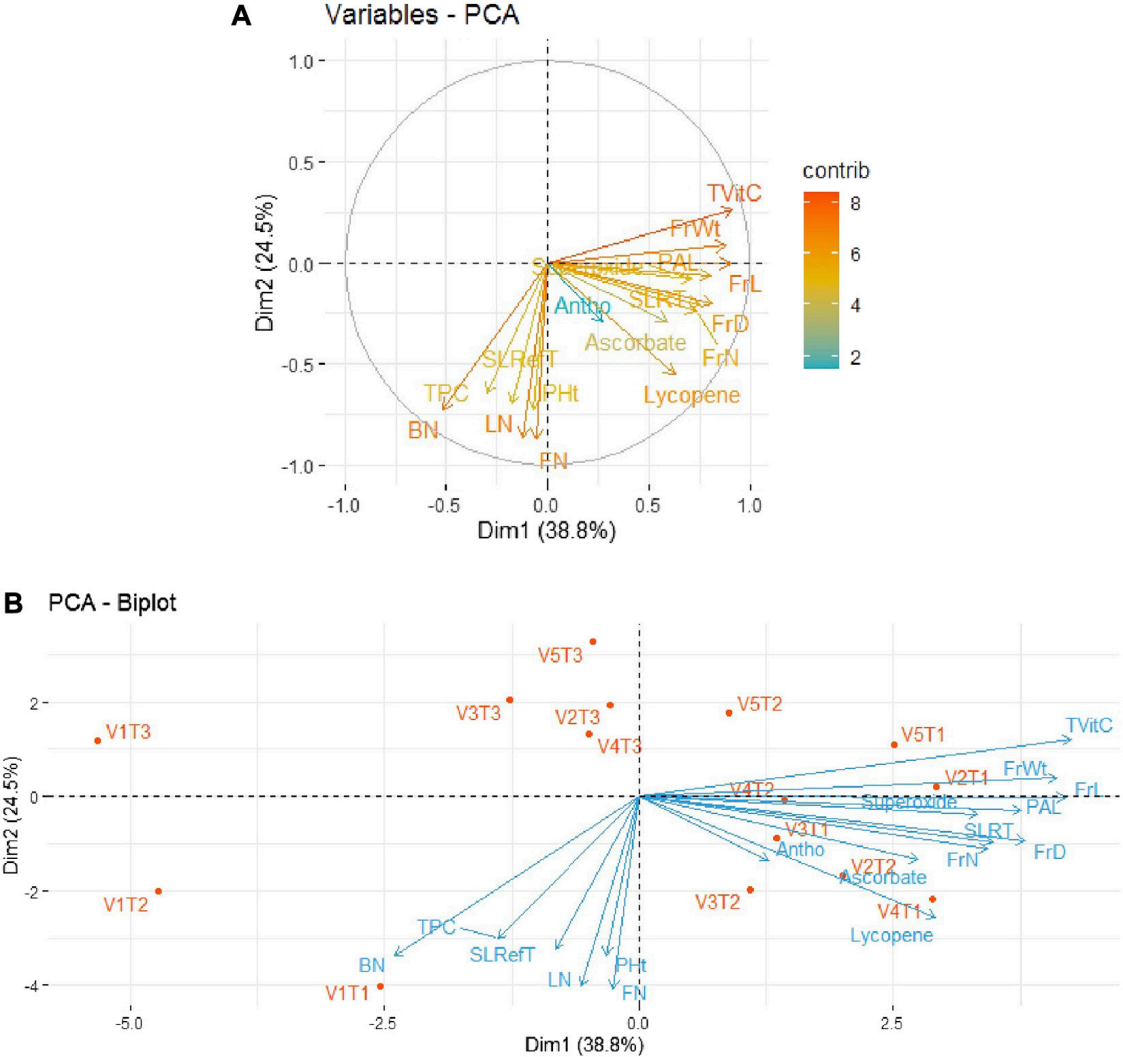
**(A)** Individual PCA plot of variables (parameters) showing the first two principal components explaining 63.3% of the variation for the year 2018–2019. **(B)** PCA biplot showing the first two principal components explaining 63.3% of the variation along with the different parameters under study for the year 2018–2019.

In order to minimize the dimensionality of the data under study, i.e., five tomato genotypes and 17 traits, in an interpretable way so as to retain most of the information, principal component analysis (PCA) was performed. The results for the period 2018–19 revealed that the first two principal components could explain 63.3% of the total variability among all the parameters of tomato varieties/genotypes. As depicted in Figure 3A, out of 17 traits, the first quadrant contained the parameters Tot Vit C, FrW, and PAL with the maximum contributions. The third quadrant contained the traits BN, LN, and FN as significant contributors, while the fourth quadrant contained the traits FrL, FrD, FrN, and Ly. The rest of the traits scattered in quadrants I, III, and IV remained non-significant contributors to the variability. Further, as shown in Figure 3B, the bi-plot analysis revealed the association patterns among the genotypes, traits, and treatments applied. Thus, PAL and SOD expressed a strong positive association with the variety Pusa Sheetal under the inorganic treatment (V_4_T_2_). However, another important trait, Ly, expressed a strong association with the variety Pusa Sheetal under the organic treatment (V_4_T_1_).

**FIGURE 4:**
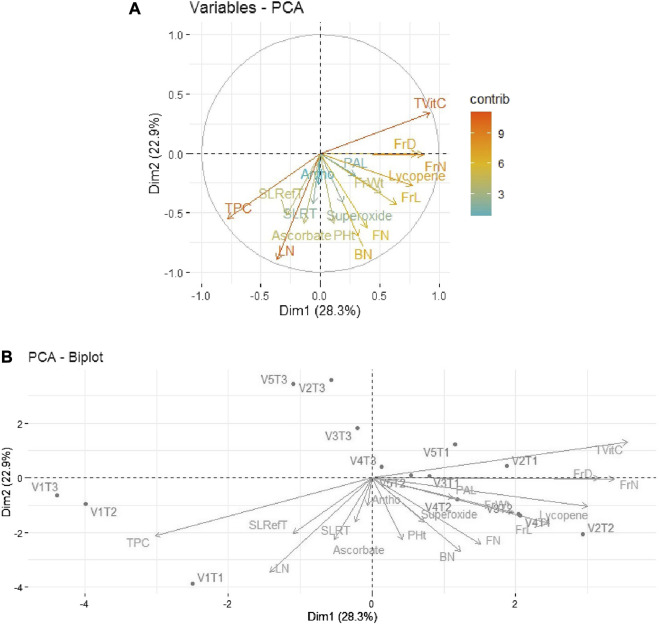
**(A)** Individual PCA plot of variables (parameters) showing the first two principal components explaining 51.2% of the variation for the year 2019–2020. **(B)**, PCA biplot showing the first two principal components explaining 51.2% of the variation along with the different parameters under study for the year 2019–2020.

Similarly, in 2019–20, 51.2% of the total variability was expressed by the first two principal components. As shown in Figure 4A, out of 17 traits, the first quadrant contained the parameter Tot Vit C with the maximum contributions. The third quadrant contained the traits TPC and LN as significant contributors to the variability, while in the fourth quadrant, moderate contributions were observed by the traits FrL, FrD, and Ly. The rest of the traits scattered in quadrants I, III, and IV remained non-significant contributors to the variability. Further, as depicted in Figure 4B, the bi-plot analysis revealed the association patterns between the genotypes, traits, and treatments applied. Thus, Tot Vit C, FrD/FrN, and Ly expressed strong positive associations with V_2_T_1_ (Avinash-3, organic), V_3_T_1_ (Pusa Rohini, organic), and V_4_T_1_ (Pusa Sheetal, organic), respectively.

Thus, it is evident that Tot Vit C is identified as a major contributor to variability.

## Discussion

### Analysis of variance

The analysis of variance in Table 3 shows that the varietal differences, the use of organic-inorganic nutrient sources, and environmental variations significantly affected tomato shelf life under both room and refrigerated temperature conditions. Such findings have also been reported earlier by several others (Evgenidis et al., 2011; Svetlana et al., 2012; Rai et al., 2017).

### Effect of different factors on the keeping quality of tomatoes

The shelf life of tomatoes at room temperature was significantly impacted by both varieties and treatments (Table 3). The average number of days that tomato fruits took to wrinkle was better in the organic environment than in the inorganic environment. All the genotypes had maximum shelf life when grown in an organic environment. However, Pusa Sheetal had a maximum shelf life of 8.35 days when grown in an organic environment, showing an increase of 12% over the control (Table 2; Figure 1). The Swaraksha genotype did not reflect any different behavior in terms of keeping quality as compared to the organic vs inorganic environment. Overall, organic produce yielded better keeping quality at room temperature.

The shelf life of tomatoes at refrigerated temperatures was significantly impacted by varieties and their interactions with treatments (Table 3). Shelf life was improved in the organic nutrient environment as compared to the inorganic environment for all genotypes. A maximum storage shelf life of 14.80 days was documented in the Pusa Sheetal tomato variety, followed by Angoorlata (13.16 days) and Pusa Rohini (11.17 days), as presented in Table 2 and Figure 2.

Numerous studies confirm that tomatoes grown in an organic environment have a higher vitamin C content than those grown in an inorganic environment (Lundegardh and Martensson, 2003; Yadav et al., 2020). Organic farming does not use nitrogen-based fertilizers, and, as a result, plants respond by activating their own defense mechanisms, increasing the levels of all antioxidants. Vallverdu et al. (2012) have pointed out that plants suffering from extra stress produce additional polyphenols. Oliveira et al. (2013) have also stated that tomato fruits obtained from organic farming realize stress conditions that result in oxidative stress and the deposition of increased concentrations of emulsifiable solids such as sugars and other admixtures that contribute to the nutritional qualities of the fruit, such as vitamin C and polyphenol compounds. Regarding the shelf life of tomatoes, a non-significant difference in the days to fruit decomposition under storage could also be ascribed to the lack of difference in the chemical composition of the different treatments, as reported by Abolusoro et al. (2017). Thus, the likely reason for better shelf life could be attributed to better growth resulting in firmer fruits with greater pericarp thickness as an explanation for proper and sufficient availability of all macro- and micronutrients (Gosavi et al., 2010). Akand et al. (2015) showed tha**t** organic nutrients had a significant influence on the shelf life of tomatoes under different storage conditions, namely, tomatoes sprayed at room temperature, tomatoes kept in polyethylene bags at room temperature, and tomatoes kept in polyethylene bags at refrigerated conditions. In another study, Laxmi et al. (2015) also reported an increase in the shelf life of tomatoes with the application of vermicompost.

Thus, it can be concluded that the fruits of Pusa Sheetal tomatoes grown under organic conditions have the longest shelf life at refrigerated temperatures.

### Coefficient of variation, heritability, and genetic advance

The shelf life of tomatoes at room temperature with a range (5–9.99) and mean (7.12) expressed considerable variations as a genotypic coefficient (12.54%), phenotypic coefficient (15.57%), coefficient of variation (12.91%), heritability in the broad sense (64%) and genetic advance (21.18%) as presented in Table 2. However, the shelf life of tomato fruit at refrigerated temperature with a range (6–16) and mean (10.74) expressed considerable genotypic coefficient of variation (20.59%), phenotypic coefficient of variation (21.93%), coefficient of variation (12.04%), broad sense heritability (86%) and genetic advance (39.95%) as presented in Table 2.

The high genotypic and phenotypic coefficients of variation observed indicate that these characters are less susceptible to environmental instability. Hence, more importance should be given to these characteristics when selecting genotypes from the current material to be utilized in future crop improvement programs. High estimates of heritability do not always mean high genetic gain. Johnson et al. (1955) advocated that heritability and the genetic advance values together would give a striking picture for an efficient judgment in forecasting the expected impact of selection. High heritability in conjugation with higher genetic advance values was documented for most of the characters under study and can be given due importance as desirable parameters in the selection process for improvement, and this can be caused by additive gene action. Thus, improvement can be practiced without progeny testing. The higher estimates of phenotypic than genotypic coefficients of variation indicate that the conspicuous variation was not only caused by the genotypes alone but also by the environment.

Thus, the shelf life of tomatoes at refrigerated temperatures can be safely considered when selecting Pusa Sheetal as a novel genotype source suitable for organic farming.

## Association ship between diverse traits including their direct and indirect effects on tomato shelf life

Correlation coefficient analysis helps to understand the nature and magnitude of the interaction between different quantitative traits to determine the component traits on which selection can be based for genetic improvement in the target trait of shelf life. The pooled analysis of both periods studied for the shelf life of tomato fruits stored at room temperature revealed positive and significant phenotypic correlation coefficient values with FrL (0.36), FrW (0.50), Ly (0.36), refrigerated temperature (0.55), An (0.33), and PAL (0.41). However, the pooled analysis of both periods studied for the shelf life of tomato fruits stored under refrigerated conditions revealed highly significant and positive correlation coefficient values with traits such as PHt (0.28), LN (0.46), TPC (0.48), and An (0.32), but a negative and significant correlation with FrN (−0.32) and Tot Vit C (−0.40). In general, for almost all characteristics, the genotypic correlation coefficient values for tomato shelf life in room and refrigerated temperatures were found to be higher than their respective phenotypic values.

The relationships between diverse traits varied for the PH, NB, NL, NF, FrW, TPC, An, and SOD reflected as color gradients in heat maps indicating their contributions towards shelf life (Supplementary Figures S1–S3, respectively).

The Table 4 revealed direct and indirect impacts of diverse traits on storage life of tomatoes stored at room temperature was impacted maximum directly in a positive way by the trait SL Ref T followed by FrW, Tot Vit C, PAL, NB, FrL, PH and AP. However, the trait FrD contributed maximum direct negative effect followed by Ly, An, NL, TPC, NF, SOD and NFr. On the other hand, Table 5 indicated the direct and indirect impacts on the genotypic correlation of shelf life of tomato fruits stored at refrigerated temperature was maximum influenced directly in a positive way by the trait An followed by FrD, Ly, SL RT, and NL. However, the trait FrW contributed maximum direct negative effect followed by PAL, SOD, PH, and NF.

Thus, from the findings of the correlation coefficients and the direct and indirect effects of different traits on the shelf life of tomatoes at refrigerated temperatures, it is inferred that the trait An can be taken into account when making an indirect selection of the most suitable genotype for organic cultivation.

### Principal component analysis

Principal component analysis (PCA) is an algorithm for unsupervised dimensionality in statistics. It aims to transform more correlated variables into fewer independent variables without sacrificing the characteristics of the associated variables. A summary of linear relationships between inputs and variables is given below.

1. PCA helps in dimensionality reduction by converting a set of correlated variables to non-correlated variables.
2. It finds a sequence of linear combinations of variables.
3. PCA also serves as a tool for better data visualization of high-dimensional data. We can create a heat map to show the correlation between each component.
4. PCA is often used to help deal with multicollinearity before a model is developed.
5. It highlights how the data is a good storyteller on its own.

### These models are useful for data interpretation and variable selection

Genetic variability is a prerequisite for crop improvement and plant survival in nature. Having a broader genetic base within the breeding material or the crop plays a crucial role in cultivar development programs, owing to improved diversity in offspring, which can be effectively utilized for genetic enhancements (Nimbalkar et al., 2017).

Principal component analysis (PCA) performed on the five tomato genotypes and 17 traits for the two investigated periods (2018–19 and 2019–20) showed how the first two principal components accounted for 63.3% and 51.2% of the total variability, respectively, for the years 2018–19 and 2019–20. PAL and SOD exhibited strong positive associations with the variety Pusa Sheetal under the inorganic treatment (V4T2), and Ly showed strong associations with the variety Pusa Sheetal and the organic treatment (V4T1) for the year 2018–19. For the year 2019–20, Tot Vit C, FrD/FrN, and Ly exhibited strong positive associations with V2T1 (Avinash-3, organic), V3T1 (Pusa Rohini, organic), and V4T1 (Pusa Sheetal, organic). Thus, it is evident that Tot Vit C is identified as a major contributor, and selecting genotypes based on this trait would be helpful in improving the shelf life of tomatoes. Similar findings have also been reported by many other researchers (Lavelli et al., 2001; Evgenidis et al., 2011; Svetlana et al., 2012; Rai et al., 2017; Sivakumar et al., 2020).

Based on a thorough analysis and interpretation of the available variability, heritability, genetic advance, interpersonal relationships, direct and indirect effects for various traits, genotypes, and environmental conditions are revealed. As conclusive insights, the trait “An” appears to be a promising indicator for indirect selection of the better genotype for shelf life, and the genotype Pusa Sheetal demonstrated to be a novel source for better shelf life at refrigerated temperatures. Thus, the trait “An” may be preferred during selection for shelf life and Pusa Sheetal during hybridization based crop improvement programs for organic farming and enhanced shelf life.

### Future prospects of gene editing to enhance tomato shelf life

Breeding tomatoes is extremely difficult because of the tomato’s limited genetic base. However, quick and effective tomato breeding is now achievable thanks to the development of clustered regularly interspaced short palindromic repeat (CRISPR)-associated protein 9 (CRISPR/Cas9) genome editing. The basic assumptions and key considerations of the proposed gene editing model are that CRISPR/Cas-based applications could potentially be used for plant breeding as follows.

(A) Multiplex gene knockouts, gene deletions, and indels can all be generated through CRISPR/Cas-mediated alteration.
(B) Gene stacking for multiple traits, gain-of-function correction, and gene insertion or replacement to generate novel traits for crop improvement can all be achieved through gene insertion and replacement mediated by either homology-directed repair or non-homologous end joining.
(C) Applications of base editing to improve agricultural traits include whole-gene screening, precise amino acid substitution, stop codon gene disruption, and gene regulation.
(D) Engineering of the regulatory site in the promoter, enhancer, or untranslated region using the CRISPR/Cas9 system.

“uORF” stands for upstream open reading frame. CRE stands for cis-regulatory element (Muntazir et al., 2021).

The various genome editing techniques applied to tomatoes to improve their quality and shelf life are mentioned in Table 6 and can be attempted by the following potential institutions.

• ICAR-IIVR—Indian Institute of Vegetable Research Varanasi, Uttar Pradesh, India.
• ICAR-IIHR—ICAR-Indian Institute of Horticulture Research, Bengaluru, India.
• ICAR- IARI—Indian Agricultural Research Institute, New Delhi, India.
• CSIR-NBRI—National Botanical Research Institute, Lucknow, India.
• DBT-NIPGR—National Institute of Plant and Genome Research, India.
• CSIR- CCMB—Center for Cellular and Molecular Biology, Hyderabad, Telangana, India.
• DBT-RCB—Regional Center for Biotechnology, Faridabad, Haryana, India.
• Jawaharlal Nehru University, New Delhi, India.
• University of Delhi, Delhi, India.
• University of Hyderabad, Telangana, India.

**TABLE 6:** Application of genome editing techniques in tomato to improve their quality and Shelf life.

S. No.	Species gene	Editing tools	Transformation methods	Target gene	Function of target gene	Outcomes	References
1	Tomato	CRISPR/Cas9	Agrobacterium	ALC	Inhibit ethylene synthesis (SN1 is an insertion of an actual Inhibitor gene ALC	Mutants with longer shelf life as compared to wild type	Chen et al. (2018)
			tumefaciens-mediated				
			transformation				
2	Tomato	CRISPR/Cas9	Agrobacterium	RIN	Inhibit ethylene synthesis and specific biochemical processes related to fruit ripening	Mutant lines exhibited lower ethylene contents and delayed fruit ripening	Jung et al. (2018)
		(SDN1)	tumefaciens-mediated				
			transformation				
3	Tomato	CRISPR/Cas9	Agrobacterium	ALC	Inhibit ethylene synthesis (SN2 seems to be a knockout mutant of the RIN gene	Mutants with longer shelf life as compared to wild type	Wang et al. (2017)
		(SDN2)	tumefaciens-mediated				
			transformation				
4	Tomato	CRISPR/Cas9	Not mentioned	SBP-CNR and NAC-NOR	Transcription factor of ripening genes	Mutants displayed partial non-ripening phenotypes	Gao et al. (2019)
		(SDN1)					
5	Tomato	ZFNs (SDN1)	Not mentioned	NF-Y, L1L4, NF-YB6	Responsible for biosynthesis for seed storage proteins and fatty acids	Mutants showed varied metabolite profiles and high amounts of OA as compared to wild type	Gago et al. (2017)

Modern genetic techniques like genome editing have recently been developed as an innovative way to increase the nutritional value and shelf life of tomatoes. Numerous horticulture crops can successfully introduce mutations (Insertions and Deletions) using various genome editing techniques, such as ZFNs, TALENs, and the CRISPR/Cas9 system, to address and reduce problems related to shelf life and nutritional levels. Many research institutions and universities in India are working on tomatoes to extend their shelf life and improve their quality. Narasimha Rao Nizampatnam from the University of Hyderabad, and many more are working on gene editing in tomatoes and other crops in India. In their research of 2023, Nizampatnam et al. worked on NSP1 genes that enhance the flavor of tomato mutants and used genome editing to create mutants of the tomato ripening inhibitor (RIN) gene, which extended longevity but contained fewer carotenoids. The genome-edited RIN alleles were crossed with a dominant phototropin1 mutant, which boosts carotenoids and volatiles, to raise carotenoid levels. Nizampatnam and their collaborators have used the dominant *phototropin1* mutant gene to improve carotenoids, taste, and flavor in tomato mutants/cultivars.

Genetically modified/engineered crops have already been developed by many countries, with most of them accepting and implementing the existing GMO (transgenic organisms) regulatory frameworks based on case-by-case assessments of the status of genome-edited species. However, the global regulatory environment for genome-edited organisms is much more heterogeneous than it is for traditional GMOs, as the regulatory triggers for the current bio-safety laws vary between legislations and different countries pursue different approaches to address/include genome-edited organisms. This fact was acknowledged by international organizations like the OECD (the Organization for Economic Co-operation and Development), which is attempting to harmonize the regulatory control of biotechnology. It is difficult to imagine how improved scientific collaboration and comprehension of these technologies could resolve this issue. To fully utilize genome editing technology in crop improvement programs and meet the demand for high-quality, nutrient-rich food that is accessible to the world’s expanding population, the adoption of appropriate regulations must be expedited (Eckerstorfer et al., 2019).

**FIGURE 5:**
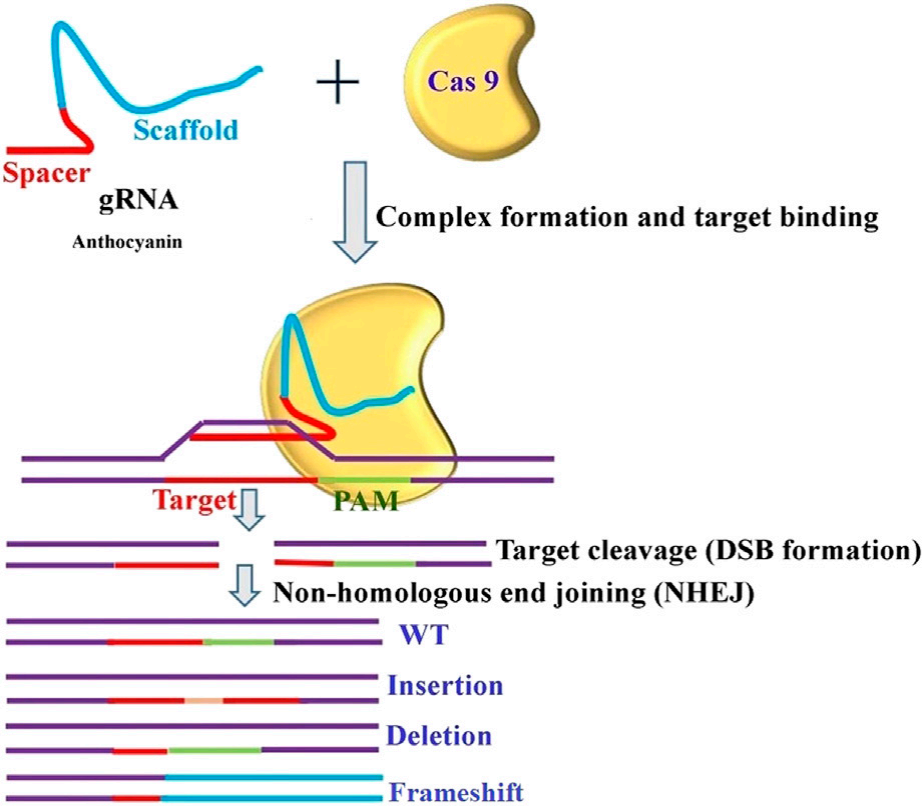
Gene editing for enhancement of tomato shelf life.

A clear, universal regulatory system (same rules for all genome-edited organisms and GMOs) that can support its widespread application with safety and public acceptance is necessary to address some of the related challenges. Very recently, researchers from the Donald Danforth Plant Science Center developed a genome editing tool that controls the transposable elements’ insertion site and cargo delivery in soybean (https://www.isaaa.org/kc/cropbiotechupdate/ged/article/default.asp?ID=20104). The CRISPR/Cas9 system has accelerated the speed of research projects by providing easy, efficient, and precise approaches to genome editing. In addition, CRISPR/Cas9 is no longer just a pair of scissors for cleaving the DNA of the genome. It can change one nucleotide into another and modify the epigenetic environment at the target site (Chandana et al., 2022). Several insights narrating different facets of gene editing for trait- and crop-specific manipulation have been explained by various researchers (Mahto et al., 2022; Singh et al., 2022; Gupta et al., 2023). In the majority of crops, the selection of traits to be improved, either through classical breeding or genetic alterations, was primarily driven by technology, taking into consideration the needs and benefits of farmers, processors, and distributors. Using a similar method on tomato seedlings, transient expression of Wus2, ipt, and STM facilitated the regeneration of complete tomato plants. Thus, it is possible to apply the same approach to produce gene-edited shoots by temporarily transforming the stems of soil-grown plants. Following the cultivation of prominent crops, CRISPR/Cas9 systems gained recognition for their ability to modify the expression of flowering genes in tomatoes. In this study, DNA constructs for ZFNs (Hilioti et al., 2016) were introduced into tomato seeds through electroporation. Following the demonstration of the power of genome editing technology, its successful application was mimicked in tomatoes (Zsögön et al., 2018). This also applies to vegetables, which have been continuously selected for improved shelf life and shipping quality, resulting in the production of crop varieties such as strawberry “cardboard” and bouncing tomato (Georges and Ray, 2017). To improve the quality and shelf life of tomatoes by modifying the anthocyanin nutrition component, a generalized schematic application of CRISPR technology is depicted in Figure 5.

## Conclusion

The biochemical trait Anthocyanin has proven to be potential contributors towards enhanced shelf life in tomatoes. The genotype Pusa Sheetal is identified as a novel source for better shelf life and is suitable for organic farming. Thus, anthocyanins can be targeted for quantity enhancement toincrease the shelf life of tomatoes through a genome editing approach. A generalized but trait-specific mechanism for genome editing has been proposed in this study.

## Data Availability

The original contributions presented in the study are included in the article/Supplementary Material, further inquiries can be directed to the corresponding authors.

## References

[B1] Abolusoro P. F. Abolusoro S. A. Adebiyi O. T. V. Ogunremi J. F. (2017). Evaluation of different manures application on fruit quality of tomato in the derived savannah ecological zone of Nigeria. Hortic. Int. J. 1 (2), 35‒37. 10.15406/hij.2017.01.00006

[B2] Adeniyi H. Ademoyegun O. (2012). Effect of different rates and sources of fertilizer on yield and antioxidant components of tomato (lycopersicon lycopersicum). Afr. J. 7 (2), 135–138. 10.3923/aj.2012.135.138

[B3] Akand H. Khairul Mazed H. E. M. Islam M. A. Pulok Md. Chowdhury S. N. Moonmoon J. F. (2015). Effect of organic manures on assessment of shelf life of tomato varieties (Lycopersicon esculentum Mill). IJAR 1 (5), 94–97.

[B4] Allard R. W. (1960). Principles of plant breeding. New York: John Willey and Sons Inc.

[B5] Bhowong A. Stout M. J. Attajarusit J. Tantasawat P. (2009). Defensive role of tomato polyphenol oxidases against cotton bollworm (Helicoverpa armigera) and beet armyworm (Spodoptera exigua). J. Chem. Ecol. 35, 28–38. 10.1007/s10886-008-9571-7 19050959

[B6] Chandana B. S. Mahto R. K. Singh R. K. Ford R. Vaghefi N. Gupta S. K. (2022). Epigenomics as potential tools for enhancing magnitude of breeding approaches for developing climate resilient chickpea. Front. Genet. 13, 900253. 10.3389/fgene.2022.900253 35937986 PMC9355295

[B7] Charul S. Kumar R. Sehgal H. Bhati S. Singhal T. Gayacharan (2023). Unclasping potentials of genomics and gene editing in chickpea to fight climate change and global hunger threat. Front. Genet. 14, 1085024. 10.3389/fgene.2023.1085024 37144131 PMC10153629

[B8] Chen Y.-R. Lü P. Yu S. Zhu N. Zhou B. Pan Y. (2018). Genome encode analyses reveal the basis of convergent evolution of fleshy fruit ripening. *Nat.* Plants. 4, 784–791. 10.1038/s41477-018-0249-z 30250279

[B9] Dewey D. R. Lu K. H. (1959). A correlation and path-coefficient analysis of components of crested wheatgrass seed production ^1^ . Agron. J. 51 (9), 515–518. 10.2134/agronj1959.00021962005100090002x

[B10] Eckerstorfer M. F. Engelhard M. Heissenberger A. Simon S. Teichmann H. (2019). Plants developed by new genetic modification techniques-comparison of existing regulatory frameworks in the EU and non-EU countries. Front. Bioeng. Biotechnol. 7, 26. 10.3389/fbioe.2019.00026 30838207 PMC6389621

[B11] Evgenidis G. Traka-Mavrona E. Koutsika-Sotiriou M. (2011). Principal component and cluster analysis as a tool in the assessment of tomato hybrids and cultivars. Hindawi Publ. Corp. Int. J. Agro. 10.1155/2011/697879

[B12] FAOSTAT (2020). FAOSTAT [WWW document]. Available at: http://www.fao.org/faostat/en/#data (accessed June 18, 2020).

[B13] Gago C. Drosou V. Paschalidis K. Guerreiro A. Miguel G. Antunes D. (2017). Targeted gene disruption coupled with metabolic screen approach to uncover the LEAFYCOTYLEDON1-LIKE4(l1l4) functionin tomato fruit metabolism. PlantCell Rep. 36, 1065–1082. 10.1007/s00299-017-2137-9 28391527

[B14] Gao Y. Zhu N. Zhu X. F. Wu M. Jiang C. Z. Grierson D. (2019). Diversity and redundancy of the ripening regulatory networks revealed by the fruit ENCODE and the new CRISPR/Cas9 CNR and NOR mutants. Hortic. Res. 6:39. 10.1038/s41438-019-0122-x 30774962 PMC6370854

[B15] Georges F. Ray H., (2017). Genome editing of crops: A renewed opportunity for food security. Gm. Crops Food. 8(1):1–12. 10.1080/21645698.2016.1270489 28075688 PMC5592977

[B16] Gosavi P. U. Kamble A. B. Pandure B. S. (2010). Effect of organic manures and biofertilizers on quality of tomato fruits. Asian J. Hort. 5 (2), 376–378.

[B17] Gupta A. Sharma T. Singh S. P. Bhardwaj A. Srivastava D. Kumar R. (2023). Prospects of microgreens as budding living functional food: Breeding and biofortification through OMICS and other approaches for nutritional security. Front. Genet. 14, 1053810. 10.3389/fgene.2023.1053810 36760994 PMC9905132

[B18] Hilioti Z. Ganopoulos I. Ajith S. Bossis I. Tsaftaris A. (2016). A novel arrangement of zinc finger nuclease system for *in vivo* targeted genome engineering: The tomato LEC1-LIKE4 gene case. Plant Cell. Rep. 35, 2241–2255. 10.1007/s00299-016-2031-x 27473525

[B19] Johnson H. W. Robinson H., E. Comstock R. E. (1955). Estimates of genetic and environmental variability in soybeans ^1^ . Agro. J. 47, 314–318. 10.2134/agronj1955.00021962004700070009x

[B20] JungLeeBaeKang Y. J. G.-J. S. K. K. (2018). Reduced ethylene productionin tomato fruitsupon CRSPR/Cas9-Mediated LeMADS-RIN mutagenesis. Korean *J. Hortic. Sci. Technol*. 36, 396–405. 10.12972/kjhst.20180039

[B21] Lavelli V. Pagliarini E. Giovanelli G. Peri C. Zanoni B., (2001). The antioxidant activity of tomato. I. Evaluation of fresh and processed products by chemical–physical indexes and biochemical model systems through principal component analysis. 10.17660/ActaHortic.2001.542.25

[B22] Laxmi P. R. Saravanan S. Naik M. L. (2015). Effect of organic manures and inorganic fertilizers on plant growth, yield, fruit quality and shelf life of tomato (*Solanum lycopersicon* L). Int. J. Ag. Sci. Res. 5 (2), 7–11.

[B23] Lundegardh B. Martensson A. (2003). Organically produced plant foods - evidence of health benefits. J Soil and Plant Sci. 53 (1), 3–15. 10.1080/09064710310006490

[B24] Luthria D. Singh A. P. Wilson T. Vorsa N. Banuelos G. S. Vinyard B. T. (2010). Influence of conventional and organic agricultural practices on the phenolic content in eggplant pulp: Plant-to-plant variation. Food Chem. 121, 406–411. 10.1016/j.foodchem.2009.12.055

[B25] Mahto R. K, Ambika Singh C. Chandana B. S. Singh R. K. Verma S. Gahlaut V. (2022). Chickpea biofortification for cytokinin dehydrogenase *via* genome editing to enhance abiotic-biotic stress tolerance and food security. Front. Genet. 13, 900324. 10.3389/fgene.2022.900324 35669196 PMC9164125

[B26] Miller P. A. Williamson J. C. Robinson H. F. Comstock R. E. (1958). Estimates of genotypic and environmental variances and covariances in upland cotton and their implications in selection ^1^ . Agron 50 (3), 126–131. 10.2134/agronj1958.00021962005000030004x

[B27] Muntazir M. Dar A. A. Skalicky M. Tyagi A. Bhagat N. Basu U. (2021). CRISPR-based genome editing tools: Insights into technological breakthroughs and future challenges. Genes. 12 (6), 797. 10.3390/genes12060797 34073848 PMC8225059

[B28] Nanwai R. K. Sharma B. D. Taneja K. D. (1998). Role of organic and inorganic fertilizers for maximizing wheat (*Triticum aestivum*) yield in sandy loam soils. Crop Res. 16 (2), 159–161.

[B29] Nimbalkar R. D. Katre Y. Y. Phad D. S. (2017). Genetic diversity in chickpea (*Cicer arietinum* L). Bioinfolet 14 (1), 60–63.

[B30] Nizampatnam N. Sharma K. Gupta P. Pamei I. Sarma S. Sreelakshmi Y. (2023). Introgression of a dominant phototropin1 mutant superenhances carotenoids and boosts flavor-related volatiles in genome-edited tomato RIN mutants. bioRxiv. 10.1101/2023.05.05.539534 38151719

[B31] Oliveira A. B. Moura C. F. Gomes-Filho E. Marco C. A. Urban L. Miranda M. R. (2013). The impact of organic farming on quality of tomatoes is associated to increased oxidative stress during fruit development. PLoS One 8 (2), e56354. 10.1371/journal.pone.0056354 23437115 PMC3577808

[B32] Olsen S. R. Cole C. V. Watenabe F. S. Dean L. A. (1954). Estimation of available phosphorus in soils by extraction with sodium bicarbonate. Washington, DC: U.S. Govt. Printing Office.

[B33] Panse V. G. Sukhatme P. V. (1978). Statistical methods for agricultural workers. New Delhi: ICAR. Publication.

[B34] Perur N. G. Subramaniam C. K. Mike G. A. R. Ray H. E. (1973). Soil Fertility evaluation to some Indian farmers. Bangalore, India: U. S Agency for International Development.

[B35] Pinela J. Barros L. Carvalho A. M. Ferreira L. C. (2012). Nutritional composition and antioxidant activity of four tomato (Lycopersicon esculentum L) farmer' varieties in Northeastern Portugal homegardens. Food. Chem. Toxicol. 50, 829–834. 10.1016/j.fct.2011.11.045 22154854

[B36] Rai A. K. Vikram A. Pal S. (2017). Genetic characterization of tomato (*Solanum lycopersicum* L) germplasm for yield and quality traits through principal component analysis. Res. J.of Agri. Sci. 8 (5), 1171–1174.

[B37] Rembiałkowska E. (2007). Quality of plant products from organic agriculture. J. Sci. Food Agric. 87, 2757–2762. 10.1002/jsfa.3000

[B38] Robinson H. F. Comstock R. E. Harvey P. H. (1949). Estimates of heritability and the degree of dominance in corn. Agron. J. 41 (8), 353–359. 10.2134/agronj1949.00021962004100080005x

[B39] SAS Institute Inc (1999). SAS/QC® user's guide. Cary, NC: SAS Institute Inc.

[B40] Shankar S. K. Sumathi S. Shankar M. Usha Rani K. Reddy N. N. (2012). Comparison of nutritional quality of organically versus conventionally grown tomato. Indian J. Hort. 69 (1), 86–90.

[B41] Simova-Stoilova L. Demireveska K. Petrova T. Tsenov N. Feller U. (2006). Antioxidative protection in wheat varieties under severe recoverable drought at seedling stag. Plant Soil Environ. 54 (12), 529–536. 10.17221/427-pse

[B42] Singh R. K. Singh C. AmbikaChandana B. S. Mahto R. K. Patial R. Gupta A. (2022). Exploring chickpea germplasm diversity for broadening the genetic base utilizing genomic resourses. Front. Genet. 13, 905771. 10.3389/fgene.2022.905771 36035111 PMC9416867

[B43] Sivakumar J. Prashanth J. E. P. Rajesh N. Reddy S. M. Pinjari O. B., (2020). Principal component analysis approach for comprehensive screening of salt stress-tolerant tomato germplasm at the seedling stage. J. Biosci. 45, 141. 10.1007/s12038-020-00111-9 33361632

[B44] Sivasubramanian S. Menon M. (1973). Heterosis and inbreeding depression in rice. Madras Agric. J. 60, 1139.

[B45] Stephen O. David A. A. Abdullahi A. B. Oudare O. A. (2014). Effect of NPK and poultry manure on growth, yield and proximate composition of three Amaranthus. J. Bot. 10.1155/2014/828750

[B46] Subbiah B. V. Asija C. L. (1956). Rapid procedure for determination of Available Nitrogen in soils. Curr. Sci. 25, 259–262.

[B47] Svetlana G. Adam T. Aleksandra T. Zdravko Š. Jelica G. Janko Č. (2012). Principal component analysis of tomato genotypes based on some morphological and biochemical quality indicators. Ratar. Povrt. 49 (3), 296–301. 10.5937/ratpov49-2452

[B48] Vallverdú Q. A. Jáuregui O. Medina R. A. Lamuela-Raventós R. M. (2012). Evaluation of a method to characterize the phenolic profile of organic and conventional tomatoes. J. Agric. Food Chem. 60 (13), 3373–3380. 10.1021/jf204702f 22380972

[B49] Walkley A. J. Black I. A. (1934). Estimation of soil organic carbon by the chromic acid titration method. Soil Sci. 37, 29–38. 10.1097/00010694-193401000-00003

[B50] Wang B. Yu Q. Li N. Tang Y. Yang S. Yang T. (2017). CRISPR/Cas9-Induced targeted mutagenesis and gene replacement to generate long-shelf life tomato lines. Sci. Rep. 7, 11874. 10.1038/s41598-017-12262-1 28928381 PMC5605656

[B51] Wang Q. Xia M. Liu C. Guo H. Ye Q. Hu Y. (2008). Cyanidin-3-O-*β*-glucoside inhibits iNOS and COX-2 expression by inducing liver X receptor alpha activation in THP-1 macrophages. Life Sci. 83 (5-6), 176–184. 10.1016/j.lfs.2008.05.017 18619979

[B52] Wright S. (1960). Path coefficients and path regressions: Alternative or complementary concepts? Biometrics 16 (2), 189–202. 10.2307/2527551

[B53] Wright S. (1921). Systems of mating. I. The biometric relations between parent and offspring. Genetics 6, 111–123. 10.1093/genetics/6.2.111 17245958 PMC1200501

[B54] Yadav R. Verma S. B. Ramawat N. Yadav R. K. Aski M. Kumar R. (2020). Identification of Tomato genotypes for organic cultivation and better shelf life underNorth Indian plain condition. Ind. J. Agri. Sci. 90 (10), 157–163. 10.56093/ijas.v90i10.107980

[B55] Zsögön A. Čermák T. Naves E. Notini M. M. Edel K. H. Weinl S. (2018). *De novo* domestication of wild tomato using genome editing. Nat. Biotechnol. 36, 1211–1216. 10.1038/nbt.4272 30272678

