# Supplementary figures and images for: Potentials of genotypes, morpho-physio-biochemical traits, and growing media on shelf life and future prospects of gene editing in tomatoes
Source: Front Genome Ed. 2023 Aug 23;5:1203485. doi: 10.3389/fgeed.2023.1203485 (PMC10481343; doi:10.3389/fgeed.2023.1203485)

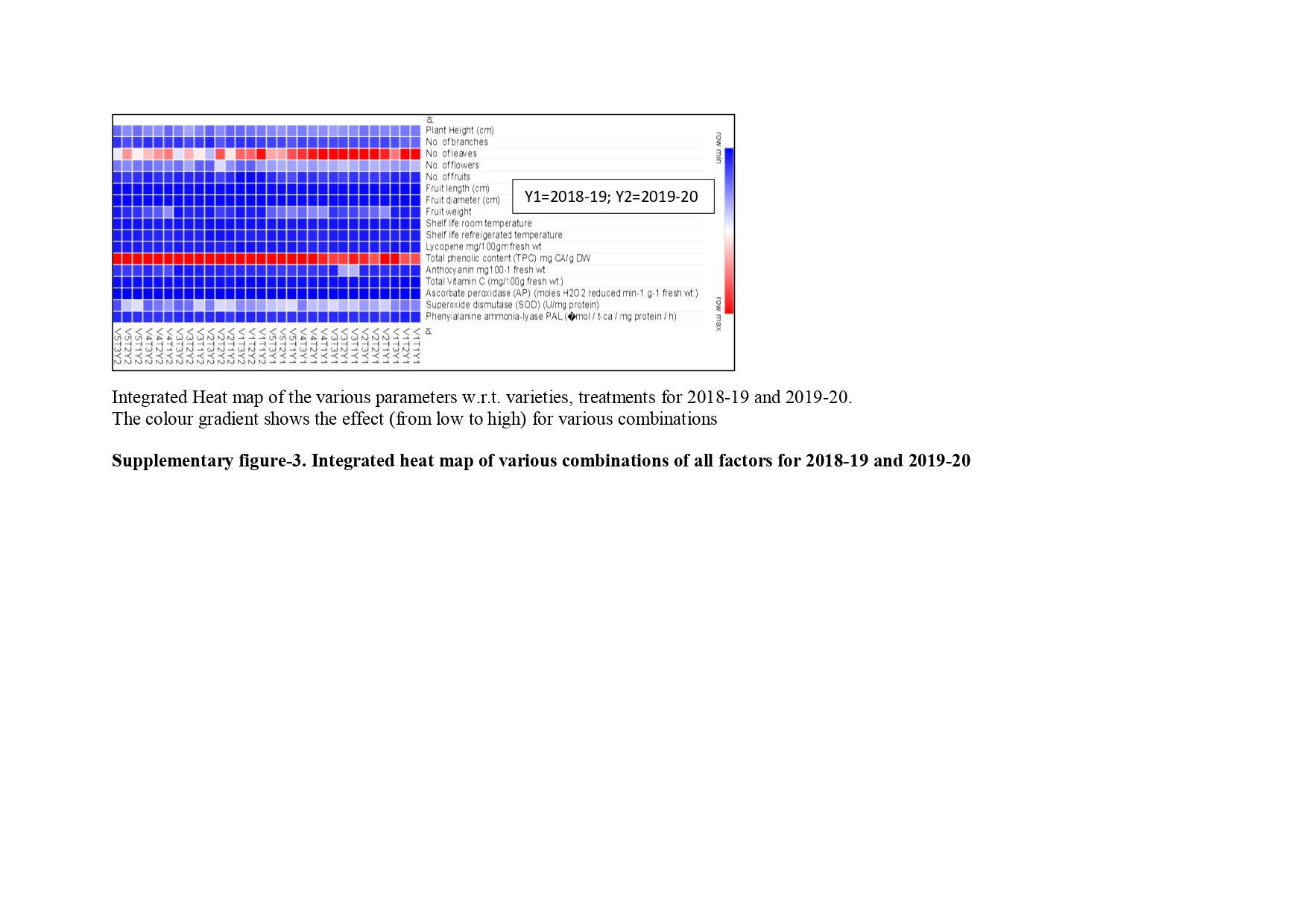

Supplement: Supplementary file 1 [file Image3.jpg]

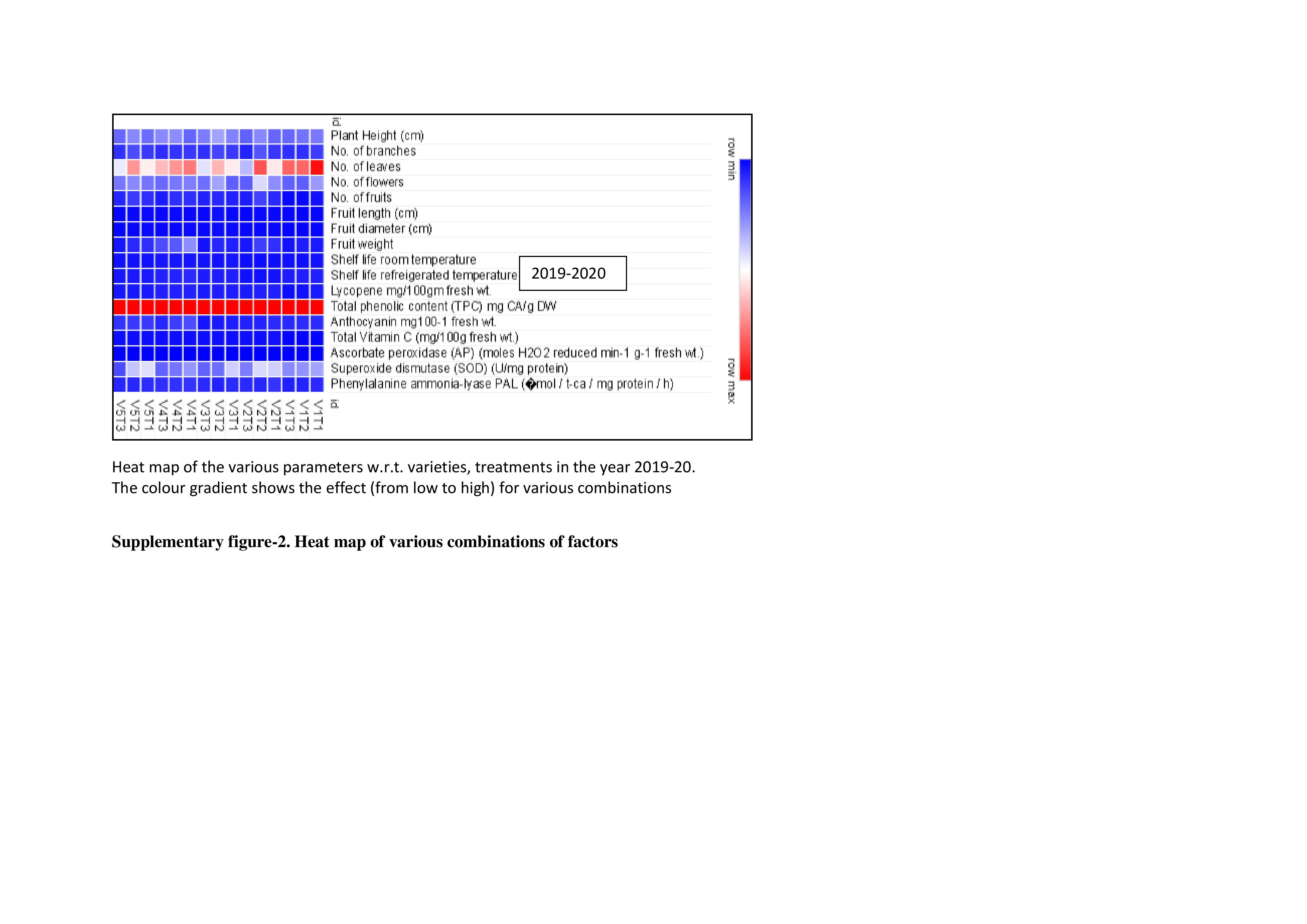

Supplement: Supplementary file 2 [file Image2.jpg]

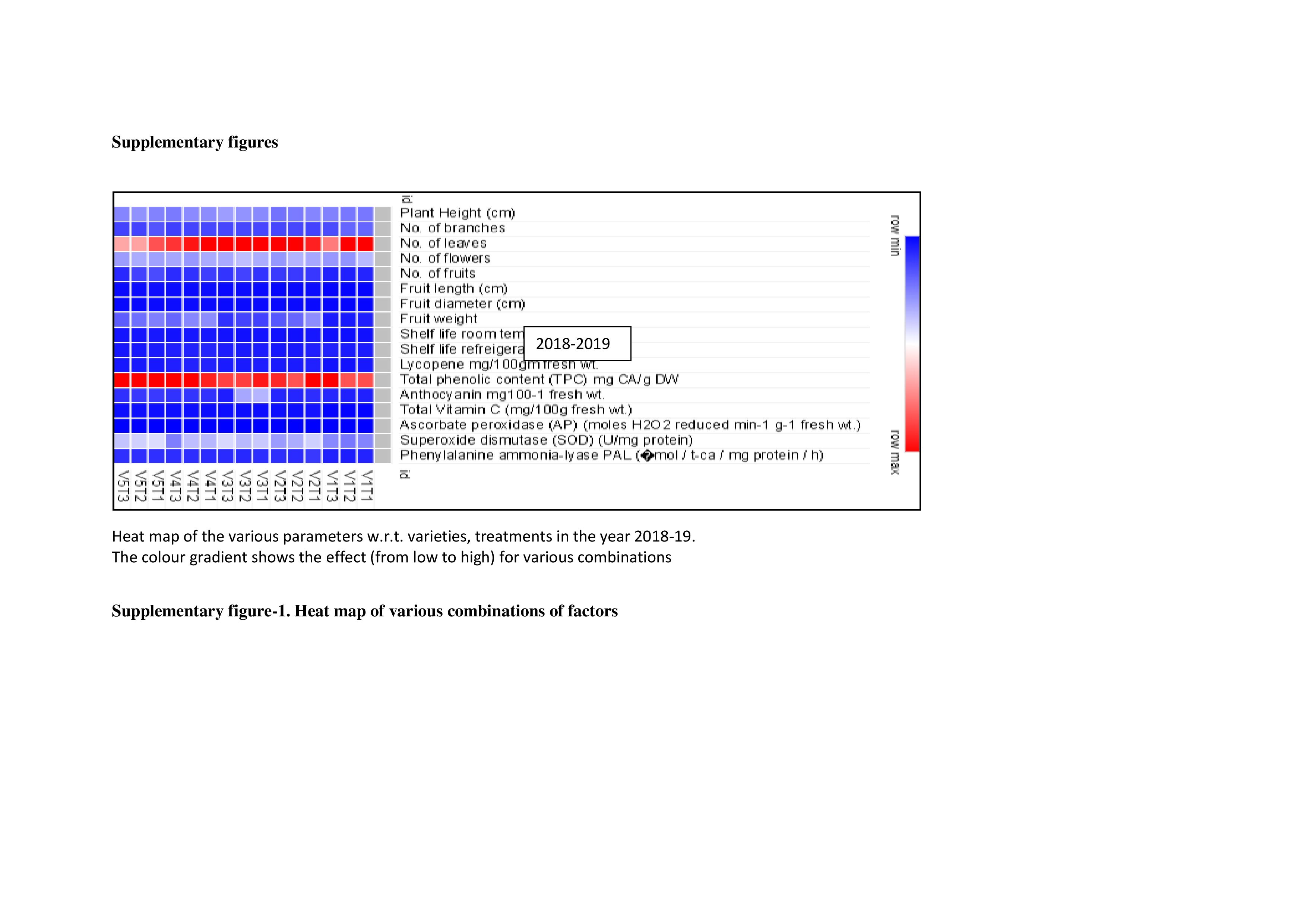

Supplement: Supplementary file 3 [file Image1.jpg]
